# Prediction of Clinically Relevant Safety Signals of Nephrotoxicity through Plasma Metabolite Profiling

**DOI:** 10.1155/2013/202497

**Published:** 2013-05-21

**Authors:** W. B. Mattes, H. G. Kamp, E. Fabian, M. Herold, G. Krennrich, R. Looser, W. Mellert, A. Prokoudine, V. Strauss, B. van Ravenzwaay, T. Walk, H. Naraoka, K. Omura, I. Schuppe-Koistinen, S. Nadanaciva, E. D. Bush, N. Moeller, P. Ruiz-Noppinger, S. P. Piccoli

**Affiliations:** ^1^PharmPoint Consulting, Poolesville, MD 20837, USA; ^2^BASF SE, Experimental Toxicology and Ecology, Z 470, D-67056 Ludwigshafen, Germany; ^3^Metanomics GmbH, Tegeler Weg 33, 10589 Berlin, Germany; ^4^Drug Safety Research Laboratories, Astellas Pharma Inc., Osaka, Japan; ^5^AstraZeneca R&D, Innovative Medicines Personalised Healthcare & Biomarkers, Science for Life Laboratory, Box 1031, 17121 Solna, Sweden; ^6^Pfizer R&D, Compound Safety Prediction, Groton, CT 06340, USA; ^7^Drug Safety Executive Council, Needham, MA 02494, USA; ^8^Metanomics Health GmbH, Tegeler Weg 33, 10589 Berlin, Germany; ^9^Analytical and Bioanalytical Development, Bristol-Myers Squibb, Princeton, NJ 08543, USA

## Abstract

Addressing safety concerns such as drug-induced kidney injury (DIKI) early in the drug pharmaceutical development process ensures both patient safety and efficient clinical development. We describe a unique adjunct to standard safety assessment wherein the metabolite profile of treated animals is compared with the MetaMap Tox metabolomics database in order to predict the potential for a wide variety of adverse events, including DIKI. To examine this approach, a study of five compounds (phenytoin, cyclosporin A, doxorubicin, captopril, and lisinopril) was initiated by the Technology Evaluation Consortium under the auspices of the Drug Safety Executive Council (DSEC). The metabolite profiles for rats treated with these compounds matched established reference patterns in the MetaMap Tox metabolomics database indicative of each compound's well-described clinical toxicities. For example, the DIKI associated with cyclosporine A and doxorubicin was correctly predicted by metabolite profiling, while no evidence for DIKI was found for phenytoin, consistent with its clinical picture. In some cases the clinical toxicity (hepatotoxicity), not generally seen in animal studies, was detected with MetaMap Tox. Thus metabolite profiling coupled with the MetaMap Tox metabolomics database offers a unique and powerful approach for augmenting safety assessment and avoiding clinical adverse events such as DIKI.

## 1. Introduction

The quality of life and human health has been dramatically improved in the past 100 years due to innovations in the fields of medicine and public health. A significant component of these innovations has been the advances in pharmaceutical treatment and prevention of disease, an early example being the dramatic treatment of streptococcal infections with the drug sulfanilamide [1]. Yet a tragic series of deaths due to kidney failure caused by a preparation of this very drug underscored the critical need to evaluate the safety of medicines prior to widespread human usage and ushered in the age of governmental oversight of pharmaceutical development [2]. Safety assessment remains a critical component in the development of new medicines today and involves various types of studies conducted at several points in the drug discovery and development process [3]. Unfortunately, this process is inefficient and suffers from significant late-stage attrition, wherein a compound once thought promising is found to be either inefficacious or have concomitant unacceptable adverse effects, that is, toxicities [4]. Even more damaging to both the business and perception of drug safety is the withdrawal from the marketplace of drugs that showed serious adverse effects after approval [5]. All in all, for the continued improvement of human health the drug development process, and in particular the safety assessment component must be improved to detect unsafe compounds at an earlier stage of their development.

Of particular importance in drug development is the assessment of a new medicine's potential for causing drug-induced kidney injury (DIKI) (or drug-induced nephrotoxicity, DIN) [6]. Not only is the kidney a vital organ, but due to its elimination of the majority of drugs and their metabolites, and its concentration of these in the process of filtration, it is particularly sensitive to chemical insults. While measurements of serum creatinine (sCr) and blood urea nitrogen (BUN) are the most widely used monitors of kidney function, they are notoriously variable and sensitive only to late stages of kidney injury [6]. It is well established that in animal models histopathological changes are observed in response to nephrotoxic compounds at doses and time points much lower than those required to produce changes in sCr and BUN [7]. Accordingly, more sensitive measures of kidney injury have been sought to improve the safety monitoring of clinical trials [7–10].

The introduction of “omics” technologies in general, where data on large numbers of distinct molecular endpoints is generated simultaneously, has provided a number of new tools promising to improve the quality of the safety assessment process [11]. By examining the effects of candidate drugs on the full tissue complement of mRNA (toxicogenomics), proteins (proteomics), or metabolites (metabolomics) subtle changes presaging overt toxicity can be detected. The latter technique has seen unique application in analyzing biofluids such as urine and blood, and as such has the capability of querying systemic perturbations in the entire organism after treatment [12]. Metabolomics has been used to identify biomarkers for disease state, drug effect and toxicity [13–16] and such biomarkers can be detected in multiple species [17, 18]. 

Metabolite profiling, like transcript profiling, is also amenable to pattern recognition approaches wherein the responses of a number of signals are collectively used to characterize a particular state or response [19]. Such pattern recognition approaches are most accurate when the reference patterns are based upon a large database of profiles collected under controlled conditions [13, 20]. Such reference databases can also serve to control for variables such as animal strain and gender used in studies [20] and assess the impact of study design [21]. Reference databases of transcript profiles have been algorithmically mined to identify transcript patterns in treated animal tissue that predict pathology seen only with more extensive treatment [22, 23], and a large collaborative effort has confirmed the validity of this approach [24].

The current investigation was proposed by the Technology Evaluation Consortium (a program under the auspices of the Drug Safety Executive Council) and undertaken to evaluate the predictive power of the MetaMap Tox metabolomics database using specific reference patterns developed based on metabolic profiles determined for data-rich reference compounds. Five archetypal pharmaceutical compounds, phenytoin, cyclosporin A, doxorubicin, captopril, and lisinopril, were selected for their diversity of chemical structure, preclinical and clinical toxicities. In particular, cyclosporin A, doxorubicin, captopril, and lisinopril all have been reported to cause kidney injury in clinical settings. Phenytoin, on the other hand, is known to cause liver, but not kidney, injury. To examine the plasma metabolome, blood was sampled at 7, 14, and 28 days from treated rats; standard clinical pathology was conducted after sacrifice at 28 days. Metabolic profiles were compared with signature profiles developed to identify or predict various toxicological modes of action (MoA). The results indicate that the preclinical and clinical adverse effects noted for these five compounds can be predicted from the matches between the metabolite profiles of the treated animals and the MoA profiles in the MetaMap Tox reference database, suggesting a unique role for metabolite profiling in safety assessment.

## 2. Materials and Methods

### 2.1. Animals and Maintenance Conditions

Wistar (CrI:WI(Han)) rats were supplied by Charles River Laboratories, Sulzfeld, Germany. The animals were housed in individual cages (floor area 800 cm²), supplied by Becker & Co., Castrop Rauxel, Germany. The animals were maintained in an air-conditioned room at a temperature of 20–24°C, a relative humidity of 30–70%, and a 12-hour light/12-hour dark cycle. Before the arrival of the animals, the room was completely disinfected (“AUTEX,” fully automatic, formalin-ammonia-based terminal disinfector, supplied by Dr. Gruß KG, Neuss, Germany). During the study, the floor and walls were cleaned weekly with a solution of 0.1% Incidin (supplied by Henkel, Düsseldorf, Germany) in water. Ground Kliba mouse/rat standard maintenance diet (cat. No. 3433) was supplied by Provimi Kliba SA, Kaiseraugst, Switzerland. The diet was assayed for chemical as well as microbiological contaminants. Drinking water was available ad libitum and regularly assayed for chemical contaminants and the presence of microorganisms. After the delivery of the rats they were accustomed to the environment and the diet for at least five days. At the beginning of the study the animals were about 10 weeks old.

### 2.2. Dosing and Experimental Design

The studies were performed according to the German Animal Welfare legislation and with the permission of the local authority (permission number 23 177-07/G08-3-001). The laboratory is AAALAC (Association for Assessment and Accreditation of Laboratory Animal Care International) certified. All compounds were administered over a period of 4 weeks to groups of 5 male and 5 female Crl:Wi(Han) rats per dose group. For each compound a group of 10 untreated males and 10 untreated females served as controls. The animals had access to water and feed ad libitum during the studies. For each compound the high dose was selected to represent the maximum tolerated dose for a 28-day study, while the low dose was selected to represent the upper limit of a human therapeutic dose. *Phenytoin* was administered daily via the food at dose levels of 600 and 2400 ppm [26]. *Doxorubicin* was administered once weekly subcutaneously at dose levels of 0.5 and 2 mg/kg bw [27]. *Cyclosporin A* was administered daily via gavage at dose levels of 20 and 45 mg/kg bw in corn oil [28–30]. *Lisinopril* was administered daily via gavage at dose levels of 40 and 400 mg/kg bw in aqueous 0.5% Carboxmethylcellulose suspension; *Captopril* was administered daily in drinking water at dose levels of 20 and 200 mg/kg bw [31, 32].

### 2.3. Blood Sampling

Between 7:30 and 10:30 h, blood samples were withdrawn from the retro-orbital sinus in all rats under isoflurane anesthesia (1.0 mL K-EDTA blood on study days 7, 14, and 28, and an additional 1.0 mL K-EDTA blood and 0.5 mL blood without anticoagulants on study day 28) after a fasting period of 16–20 h. The hematology parameters were measured in the plasma. The other blood samples were centrifuged (10°C, 2000 ×g, 10 min) and the serum as well as the EDTA plasma was separated. Clinical chemistry parameters were measured from serum. The plasma samples were frozen at −80°C until metabolite profiling was performed. In total, 120 plasma samples were used for analysis and evaluation for each compound.

### 2.4. Clinical Examinations

All animals were checked daily for any clinically abnormal signs and mortalities. Food consumption was determined on study days 7, 14, 21, and 28. Body weight was determined before the start of the administration period in order to randomize the animals and on study days 0, 4, 7, 14, 21, and 28. At the end of the treatment period, the animals were sacrificed by decapitation under Isoflurane anesthesia.

### 2.5. Clinical Pathology

The red blood cell parameter values were measured in the K-EDTA blood taken on study day 28 with an ADVIA120 instrument, Siemens. Clinical chemistry parameters were measured on a Hitachi 917 (Roche) serum samples taken on study day 28: alanine aminotransferase, alkaline phosphatase, *γ*-glutamyltransferase, inorganic phosphate, calcium, magnesium, urea, creatinine, glucose, total protein, total bilirubin, albumin, globulins, triglycerides, and cholesterol. 

### 2.6. Metabolite Profiling

For mass spectrometry-based metabolite profiling analysis, EDTA plasma samples were extracted by a proprietary method. Three types of mass spectrometry analysis were applied to all samples: GC-MS (gas chromatography-mass spectrometry) and LC-MS/MS (liquid chromatography-MS/MS) were used for broad profiling [13], while SPE-LC-MS/MS (solid phase extraction-LC-MS/MS) was applied for the determination of catecholamine and steroid hormone levels. Proteins were removed from plasma samples by precipitation. Subsequently polar and nonpolar fractions were separated for both GC-MS and LC-MS/MS analysis by adding water and a mixture of ethanol and dichloromethane. For GC-MS analysis, the nonpolar fraction was treated with methanol under acidic conditions to yield the fatty acid methyl esters derived from both free fatty acids and hydrolyzed complex lipids. The nonpolar and polar fractions were further derivatized with O-methyl-hydroxylamine and pyridine to convert oxo-groups to O-methyl-oximes then silylated before analysis [33]. For LC-MS analysis, both fractions were reconstituted in appropriate solvent mixtures. HPLC was performed by gradient elution using methanol/water/formic acid on reversed phase separation columns. Mass spectrometric detection technology was applied which allows target and high sensitivity MRM (Multiple Reaction Monitoring) profiling in parallel to a full screen analysis (patent application 2003073464). Steroid hormones, catecholamines, and their metabolites were measured by online SPE-LC-MS/MS (Solid phase extraction-LC-MS/MS) [34]. Absolute quantification was performed by means of stable isotope-labeled standards. Metabolite changes were calculated as the ratio of the mean of metabolite levels in individual rats in a treatment group relative to mean of metabolite levels in rats in a matched control group (time point, dose level, and sex). The methods applied resulted in 225 plasma analytes for semiquantitative analysis, 167 of which were chemically identified and 58 were unknown (Supplemental Table 1 available online at http://dx.doi.org/10.1155/2013/202497).

### 2.7. MetaMap Tox Evaluation of Metabolic Profiles

Discriminating metabolite patterns for various toxicological modes of action (“MoA”s) were developed from the metabolite profiles in the MetaMap Tox database for over 500 compounds as described in van Ravenzwaay et al., 2012 [35]. Briefly, metabolite patterns correlating to specific toxicological modes of action are based on at least three different chemicals from the MetaMap Tox database, which share a common toxicological mode of action (reference compounds). After identification of the significantly changed metabolites and a consistency check through experienced toxicologists, the pattern is validated against the database: a pattern should correctly identify at least one further reference compound sharing the same mode of action which has not been used to establish the pattern. Furthermore, reference compounds in MetaMap Tox which do not share this particular toxicity should not be identified. For example, the pattern for “Thyroid indirect Liver” includes 34 distinct metabolite changes. A second set of reference compounds known to also have that toxicological MoA were used to test and validate the discriminating pattern.

The database routine of pattern ranking is a two-step process: firstly, the algorithm used in the database yields a ranking list based on similarity if the test compound metabolic profile was compared to the specific patterns in MetaMap Tox. Secondly, the similarity of the metabolite changes as well as their quality and importance for a certain toxicological mode of action is evaluated by an expert panel of experienced toxicologists to determine what may be described as “confirmed” matches. In this process, the metabolite changes are assessed with respect to the strength of the change, the biochemical importance for a certain mode of action, and similarity of metabolite changes having biomarker character.

The sex- and day-stratified heteroscedastic *t*-test (“Welch test”) was applied to compare metabolite levels of dose groups with respective controls. A significance level of *P* < 0.05 was applied. Test substance related changes in the metabolome were compared: (1) with specific metabolite patterns (i.e., characteristic metabolite changes for a toxicological mode of action) called “pattern ranking,” using a split correlation based on median *t*-values, and (2) with the entire metabolome of reference compounds, called “profile comparison,” using Spearman and Pearson correlations. Based on the reference data base, a threshold value of 0.50 for male animals and 0.60 for female animals displays approximately the 95th percentile of all correlation coefficients obtained by the profile comparison. Correlation coefficients above these values are considered as indicating a clear match between two treatments.

Based on these evaluations, the toxicological mode of action (including target organs and underlying toxicological mechanisms) of the test substance was assessed. Further reference on the development of the MetaMap Tox data base can be found in, van Ravenzwaay et al., 2007, Strauss et al., 2009, and Looser et al., 2005 [13, 20, 36].

## 3. Results

### 3.1. Phenytoin

**Figure 1:**
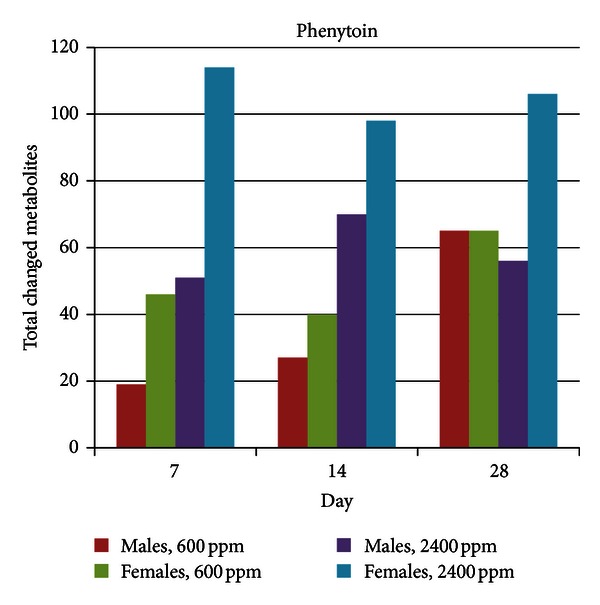
Total metabolite changes induced by phenytoin treatment. Metabolite changes were calculated as the ratio of the mean of metabolite levels in individual rats in a treatment group relative to mean of metabolite levels in rats in a matched control group (time point, dose level, and sex); the significance level was 0.05.

The metabolic profiling of phenytoin and comparison with the MetaMap Tox database has been described by Kamp et al. [25]. Briefly, however, the high dose treatment produced statistically significantly decreased body weight throughout the entire study and minimal-to to-slight centrilobular hepatocellular hypertrophy for both males and females. All other findings were minimal or within historical control ranges. On the other hand, metabolic profiling revealed a significant number of changes in metabolite levels for both dose groups and at all-time points based on a significance level of 0.05 (Figure 1). Total metabolite changes (increases and decreases) were greater for females in all groups except the low dose groups at day 28. In general, the number of metabolite changes was higher in the high dose as compared to the low dose, except for day 28 in male animals. Total metabolite changes were relatively constant over the treatment for the high dose, but increased over the course of treatment for the low dose. 

As noted in Kamp et al., the comparison of the metabolite changes induced by the high dose phenytoin treatment against the established specific metabolite patterns present in MetaMap Tox (i.e., pattern ranking) identified matches with validated patterns associated with liver enzyme induction and liver toxicity. Phenytoin did match with patterns for GABA receptor agonism; however, it is one of the reference compounds of this pattern. It might be noted that metabolic evidence for GABA receptor antagonism was present only at the low dose (which was used in the creation of this MoA pattern). However, paradoxical effects of high phenytoin doses (i.e., induction, rather than suppression, of seizures) have been noted in rats [37].

In many cases validated patterns that have confirmed matches with the metabolite profile of animals treated at one dose (e.g., higher dose) also match the metabolite profile of animals treated at the other dose (e.g., lower dose), but at a lower median correlation value that under expert evaluation cannot be “confirmed” as a match. Furthermore, in many cases several validated patterns are associated with a given mechanism of toxicity. Thus one can consider the MoA patterns from the standpoint of those that have a confirmed match at either dose, and associated with a given common mechanism. Examining the matches between the established patterns in the MetaMap Tox database and phenytoin induced metabolite profiles from this perspective shows a clear dose dependency in the number of matches for several types of liver toxicity (Table 1), giving further confidence in the prediction of liver toxicity from the metabolite profile. The phenytoin-induced metabolite profile at the high dose also produced a weak match for the pattern for “kidney, diuretic effect” but this would not necessarily be considered an adverse effect.

### 3.2. Cyclosporin A

**Figure 2:**
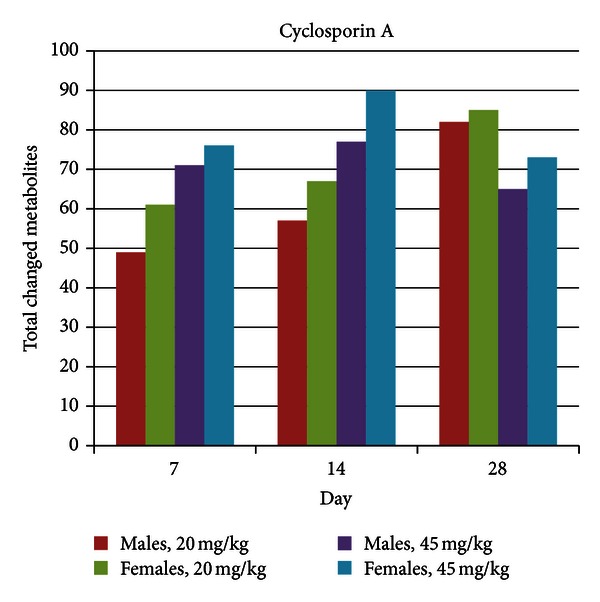
Total metabolite changes induced by cyclosporin A treatment. Metabolite changes were calculated as the ratio of the mean of metabolite levels in individual rats in a treatment group relative to mean of metabolite levels in rats in a matched control group (time point, dose level, and sex); the significance level was 0.05.

While body weight for female animals was unaffected by treatment at either cyclosporin A dose levels, that of male animals was significantly decreased in the low dose group at day 28 (−15%), and throughout the study for the high dose group (−13% on day 28). Clinical pathology and histopathology were not conducted. Metabolic profiling revealed a significant number of changes in metabolite levels for both dose groups and at all time points based on a significance level of 0.05 (Figure 2). Total metabolite changes (increases and decreases) were slightly greater for females compared to males, and higher for the high dose treatment as compared to the low dose treatment, except for day 28. In the low dose group total metabolite changes slightly increased over the course of the treatment. 

The comparison of the metabolite changes induced by the high dose cyclosporin A treatment against the established MetaMap Tox patterns (i.e., pattern ranking) identified matches with validated patterns associated with kidney toxicity, liver toxicity, and anemia (Supplemental Figure 1). Matches were also observed for patterns associated with effects on the spleen. Pattern ranking of the metabolite changes induced by the low dose cyclosporin A treatment yielded weak matches for paracetamol-derived liver toxicity (Supplemental Figure 2). Matches with the pattern for immunosuppression take into account that the low dose treatment of cyclosporin A was used in establishing that MoA pattern. Overall, the pattern ranking matches show that while signals for liver toxicity are suggested at even the low dose of cyclosporin, signals of kidney toxicity predominate at the high dose (Table 2).

### 3.3. Doxorubicin

**Figure 3:**
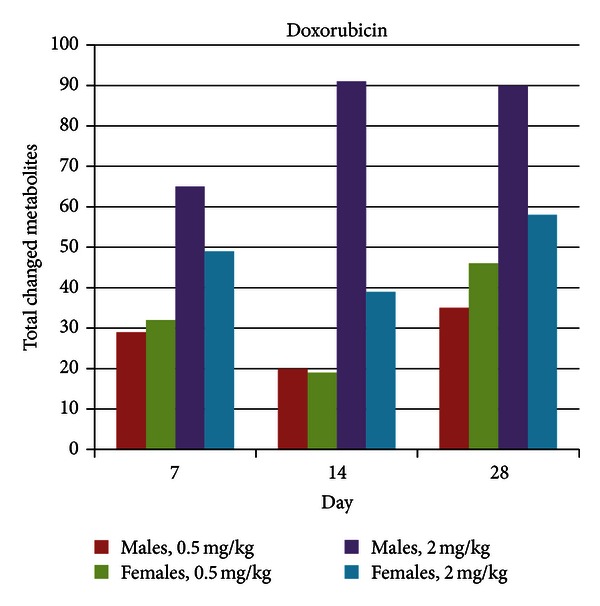
Total metabolite changes induced by doxorubicin treatment. Metabolite changes were calculated as the ratio of the mean of metabolite levels in individual rats in a treatment group relative to mean of metabolite levels in rats in a matched control group (time point, dose level, and sex); the significance level was 0.05.

Body weight was statistically significantly decreased in female animals at 28 days at the high dose level (−5%), but unaffected in males at the high dose and in either sex at the low dose level. Food consumption was significantly decreased in both sexes in the high dose group throughout the administration period. Also, in the high dose group of both sexes, total protein, albumin and globulin levels were decreased at day 28, and creatinine values and alkaline phosphatase (ALP) activities were also lower compared to controls. Glucose and inorganic phosphate levels were increased in rats of both sexes; cholesterol levels were higher only in males. Platelet counts were increased in both sexes, while red blood cell counts, hemoglobin, and hematocrit values were decreased. White blood cell (WBC) counts were markedly decreased only in males. All animals in the low dose group showed no changes in body weight or food consumption, and had no clinical pathology findings. Metabolic profiling revealed a number of changes in metabolite levels for both dose groups and at all-time points based on a significance level of 0.05 (Figure 3), albeit the changes were greater in the high dose groups. Total metabolite changes (increases or decreases) were substantially greater for males compared to females in the high dose group. Moreover, for the high dose treatment, more changes were observed as compared to the low dose treatment. Modest temporal increases in the total metabolite changes occurred over the course of the treatment. 

The comparison of the metabolite changes induced by the high dose doxorubicin treatment against the established specific metabolite patterns identified matches with validated patterns associated with liver enzyme induction, liver toxicity, and indirect effects on thyroid due to increased conjugation of thyroxin, as well as antiproliferative effects (Supplemental Figure 3). Weaker matches were observed for patterns associated with anemia and steroid biosynthesis inhibition in the adrenal cortex (only on day 28). Pattern ranking of the metabolite changes induced by the low dose doxorubicin treatment yielded a clear match with tubular necrosis in the kidney. Further weaker matches were found for bone marrow suppression (Supplemental Figure 4). Pattern matches for antiproliferative effects and bone marrow suppression must take into account that doxorubicin was used for the establishment of these patterns. The key findings are then that of signals for kidney toxicity and liver toxicity at the low dose and high dose doxorubicin treatments, respectively (Table 3).

### 3.4. Captopril

**Figure 4:**
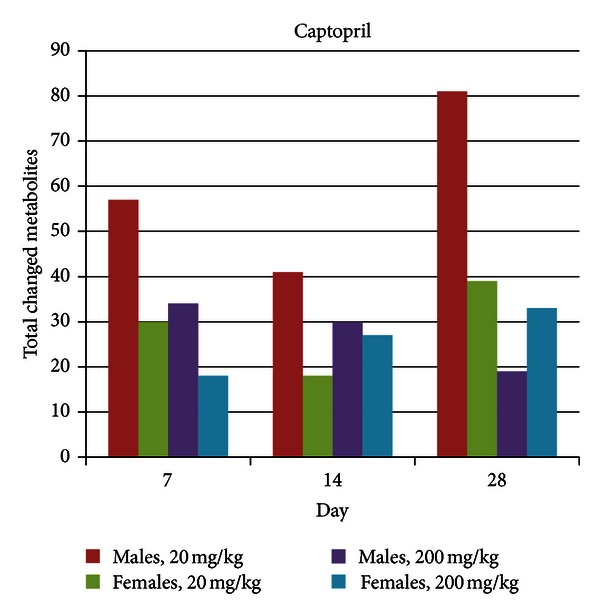
Total metabolite changes induced by captopril treatment. Metabolite changes were calculated as the ratio of the mean of metabolite levels in individual rats in a treatment group relative to mean of metabolite levels in rats in a matched control group (time point, dose level, and sex); the significance level was 0.05.

No changes in food consumption or body weight were observed in either sex at both dose levels. The only changes in clinical pathology occurred at day 28 in the high dose group, where globulins were decreased in males, and albumin levels, red blood cell (RBC) counts, and hemoglobin were decreased in females, and creatinine and total protein levels were decreased in both sexes. Metabolic profiling revealed a significant number of changes in metabolite levels for both dose groups and at all-time points based on a significance level of 0.05 (Figure 4). Interestingly enough, total metabolite changes were greatest for male rats treated with the low dose. There was not a clear temporal change in total metabolite changes in any group. 

MetaMap Tox-based pattern ranking using the metabolite changes induced by the high dose captopril treatment matches with validated patterns associated with kidney toxicity, such as glomerular-tubular defect (Supplemental Figure 5). However, the high dose treatment of captopril was used to establish this particular pattern. A further match was observed for steroid synthesis inhibition in the adrenal cortex. Pattern ranking of the metabolite changes induced by the low dose captopril treatment against the established specific metabolite patterns present in MetaMap Tox produced matches to patterns for liver and kidney toxicity as well as to those for effects on the bone marrow (Supplemental Figure 6). Weaker matches were observed for other patterns indicating effects on the kidney and liver (e.g., “glomerular-tubular defect in kidneys” and “long chain phthalates,” which is specific for a certain class of chemicals leading to peroxisomal proliferation in the liver). Thus signals for bone marrow suppression and kidney toxicity are seen at both dose levels. Recapitulating the overall Pattern Ranking graphics, liver toxicities are seen only at the low dose (Table 4). Such a paradoxical dose response has been seen for other effects of captopril, namely, the effect of this drug on drinking [38, 39], where captopril enhanced drinking at low doses but inhibited it at high doses. It is impossible to speculate if these diverse results are related.

### 3.5. Lisinopril

**Figure 5:**
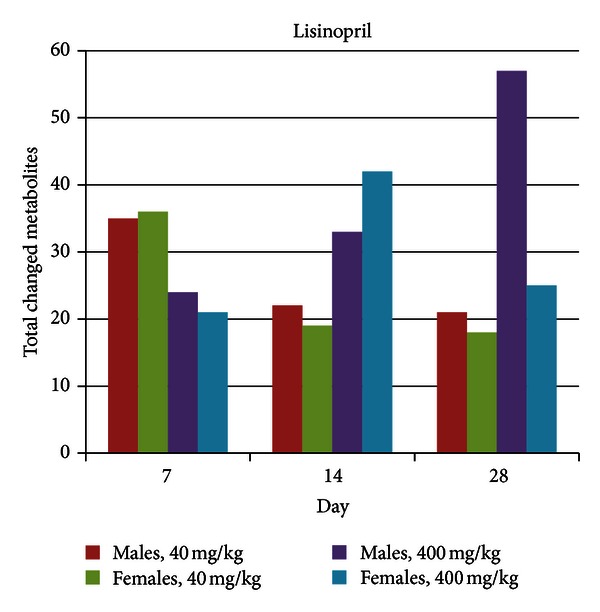
Total metabolite changes induced by lisinopril treatment. Metabolite changes were calculated as the ratio of the mean of metabolite levels in individual rats in a treatment group relative to mean of metabolite levels in rats in a matched control group (time point, dose level, and sex); the significance level was 0.05.

While food consumption was slightly decreased by Lisinopril treatment at both dose levels in both sexes and throughout the study, statistically significant body weight increases (+6%) were seen at day 28 in female animals in the high dose group. Statistically significant body weight decreases (−10% on study day 28) were seen throughout the study for male animals in the low dose group. No body weight changes were observed for males in the high dose group or females in the low dose group. Clinical pathology was seen only on study day 28, in male animals in the high dose group, where red blood cell (RBC) counts, hemoglobin and hematocrit values as well as globulin, albumin, and total protein levels were decreased. In these animals creatinine, urea, and magnesium levels were increased. Metabolic profiling revealed a significant number of changes in metabolite levels for both dose groups and at all-time points based on a significance level of 0.05 (Figure 5). In general, more changes were observed for the high dose treatment as compared to the low dose treatment. While total metabolite changes increased over the course of the study in male animals treated at the high dose, a temporal pattern was not obvious for other groups. 

MetaMap Tox-based pattern ranking using metabolite changes induced by the high dose lisinopril treatment identified matches with validated patterns associated with kidney toxicity (ACE inhibitor like) and platelet aggregation inhibition (Supplemental Figure 7). However, as with Captopril, Lisinopril was used as reference compound for the kidney toxicity patterns. Pattern ranking of the metabolite changes induced by the low dose Lisinopril treatment against the established specific metabolite patterns present in MetaMap Tox yielded weak matches with reduced feed consumption (in line with the clinical findings) in addition to that for kidney toxicity (ACE inhibitor like) (Supplemental Figure  8). Overall the metabolite profiling recapitulates the observation that while relatively few toxicities are seen at either dose, signals for platelet aggregation inhibition and kidney effects are seen at both doses (Table 5).

## 4. Discussion

The major finding from this evaluation was that the adverse effects reported in preclinical animal studies and human clinical settings for the five compounds tested in this study were detected by matches between the compound-induced metabolic profile and metabolite patterns for various toxicological modes of action (Table 6). One clear exception is that of doxorubicin-induced cardiomyopathy seen in both animals and man [27, 40], where a metabolite pattern for this toxicity had not been developed, and therefore MetaMap Tox was unable to identify such an effect. Importantly, the potential for nephrotoxicity was correctly predicted for cyclosporin A, doxorubicin, captopril, and lisinopril, albeit the latter two studies were used as reference compounds for nephrotoxicity patterns, making their nephrotoxicity prediction a fait accompli. On the other hand, phenytoin which is not reported as associated with nephrotoxicity, had no pattern matches signaling nephrotoxicity.

Interestingly, in several cases these clinically relevant adverse effects have not been reported in rodent studies. For example, phenytoin is associated with both acute and chronic liver injury in patients [41, 42] and is the third leading causative agent for acute liver failure requiring liver transplantation [43]. Other than liver enzyme induction [25, 26, 44] no such toxicity has been observed in rodent studies [45, 46]. Yet the metabolite changes induced by phenytoin treatment produced confirmed matches with several types of liver toxicity, in addition to matches to liver enzyme induction. In a similar fashion, while captopril has been associated with clinical liver injury [41, 47], no such toxicity has been reported in rats [32], with only one report of variable captopril toxicity in mice [48]. On the other hand, the metabolite profiling from rats in this study produced matches between captopril treatment and metabolite patterns associated with oxidative stress in the liver. 

Toxicities that have been reported in both preclinical and clinical studies were also identified through matches between the treatment induced metabolite profile and the mechanism-associated metabolite patterns. Cyclosporin A treatment has been reported to induce liver lesions in rat studies [49] as well as hepatotoxicity in man [50], consistent with a metabolite profile matching that of liver toxicity (Table 2). Similarly the kidney injury indicated by metabolite profiling is consistent with the nephrotoxicity well known in rodents and man [29, 51]. The anemia indicated for cyclosporin A treatment by metabolite profiling has also been identified in rodents and man [28, 52]. Anemia associated with doxorubicin treatment in both rats and man [53, 54] is also indicated in this study through metabolic profiling (Table 3), as is liver injury, seen in both rats and patients [55, 56]. Finally, the bone marrow effects indicated by metabolite profiling (Table 4) for captopril treatment have also been noted in preclinical and clinical studies [32, 57].

Some effects indicated through the metabolite profile matches have either been seen only in rat studies or have conflicting reports in the clinical literature. Thus the bone toxicity predicted by metabolite profiling for cyclosporin treatment has been noted in rats [58, 59] but does not appear to be relevant for clinical settings [60]. Similarly the platelet effects predicted for lisinopril treatment by metabolite profiling have been considered absent [61] or beneficial [62] in clinical studies. Liver enzyme induction following doxorubicin treatment has been observed in rat studies [63], consistent with the metabolite profile predictions, but has not been reported in man.

Captopril and lisinopril share a common pharmacological mechanism of action and a common pharmacophore, and as a class are note for a risk of kidney injury as noted above. However, these two compounds also have unique adverse reactions, which as noted in the results, are identified through the matches of their metabolite profile with the mechanistic patterns. In particular, a pattern for liver oxidative stress matched the metabolite profile for captopril treatment, but not that for lisinopril, in keeping with the literature recognizing liver injury as a risk for captopril [47, 64] but almost never seen for lisinopril [43].

As noted in the results, in some cases the metabolite profiles from the compound treatments in this study had already been used in the development of the metabolite pattern specific for a mechanism of action, and as such, matches between the profile and the mechanistic pattern would be expected. Such was the case for immunosuppression following cyclosporin A treatment, antiproliferative effects and bone marrow suppression following doxorubicin treatment, and the kidney toxicity following captopril or lisinopril treatment [65]. On the other hand, where a mechanistic pattern had not been developed for cardiotoxicity, no prediction of this effect could be possible even for a treatment such as doxorubin, where cardiomyopathy is the major adverse effect in both rats and humans [27, 66], albeit analysis of significantly changed individual metabolites has the potential to suggest such toxicity (data not shown).

Metabolomics has been used for over a decade as a tool to both “predict” toxicity in animals as well as understand mechanisms of toxicity [12, 14, 67, 68]. The latter application has proven valuable in elucidating areas as diverse as the effects of copper in the soil on earthworms [69] and the mechanism of testicular toxicity induced by the industrial solvent ethylene glycol monomethyl ether [70]. Prediction of toxicity from metabolomic data has been approached in several different ways. Given the complexity of NMR spectra obtained from biological fluids or tissues, the use of multivariate statistical analysis of entire spectra, broken in “bins,” has proven valuable in identifying fingerprints of treatment responses and temporal patterns [12, 19, 68]. The Consortium on Metabonomic Toxicology (COMET) project took this approach further by building a database of spectra of rat urine samples (*n* = 12935) from 80 different treatments to build a modeling system for toxicity prediction. The result was an algorithm that would detect “abnormal metabolic states” and classify them as either liver or kidney toxicity [71]. While these fingerprinting methods rely upon condensing the spectral information into a multivariate analysis (such as a principal component analysis, PCA) plot, the “loadings” of such plot data may be investigated to identify individual metabolite molecules [12]. A somewhat different approach has been taken in this project to develop the predictive patterns based on the MetaMap Tox database. A combination of GC-MS, LC-MS/MS and SPE-LC-MS/MS (Solid phase extraction-LC-MS/MS) were used to identify hundreds of unique analytes for each treatment time point. Those analytes that were consistently modulated by treatments sharing a common mechanism of action (MoA) were then used to create a predictive pattern. The plasma metabolite profile of novel treatment can then be compared against this set of MoA patterns, with the result being a number of predictions as to possible toxicities and/or physiological effects [13, 21]. 

This investigation supports the predictive power of metabolite profiles developed from a large metabolomics database and linked to physiological mechanisms of action as tools for identifying the potential of a treatment for inducing an adverse effect, importantly including nephrotoxicity. Not surprisingly, the predictions identify from the plasma of treated rats the toxicities as reported for those treatments in previous rat studies. However, the metabolite profile matches also suggest treatment-related adverse events that in some cases have been reported in clinical, but not preclinical, studies. One might speculate that the metabolite patterns might be reflective of an underlying pathophysiological response that leads to a clinical relevant adverse reaction in some humans, but is not yet phenomenologically evident in normal rats with a healthy adaptive response. For example, species differences in coumarin-induced hepatotoxicity appear to be due to differences in detoxification pathways [72]. On the other hand, environmental factors such as diet [73] or inflammation [74] may alter adaptive responses to drugs in the human population in a way that is not routinely observed in preclinical studies. Nonetheless, the promise of metabolite profiling as predictive safety assessment tool seems clear and merits further exploration and use. 

In summary, we successfully detected the key adverse events associated with five paradigm compounds (phenytoin, cyclosporin A, doxorubicin, captopril, and lisinopril) selected for their diversity of chemical structure and preclinical and clinical toxicities. Nephrotoxicity was correctly predicted for those treatments known to induce this adverse event; nephrotoxicity was not predicted for phenytoin, a negative control for this adverse effect. Additionally, MetaMap Tox provided significant additional value compared to a standard 28-day regulatory study, such as key mechanistic information, the potential to detect idiosyncratic toxicities and the differentiation of toxicities for compounds with similar chemical structure. Overall MetaMap Tox-based metabolite profiling as described here has the potential to be a powerful complement to a standard 28-day safety assessment study in rats as noted above, as well as for further qualifying final lead selection. 

## Supplementary Material

Supplementary Figures provide the detailed Pattern Ranking results for cyclosporin A, doxorubicin, captopril and lisinopril, while Supplementary Table 1 provides the measured metabolites and their fold changes for all treatment groups.

## Figures and Tables

**Table 1:** Sum of toxicity patterns matching phenytoin-induced metabolite changes.

Toxicity	Phenytoin, low dose	Phenytoin, high dose
Bone, osteoblast inhibitor	1	
CNS, GABA receptor antagonist	2	
Kidney, diuretic effect		1
Liver cholestasis		2
Liver toxicity		2
Liver, enzyme induction	1	5
Liver, paracetamol-derived toxicity		1
Thyroid, indirect effects	1	2

Grand total	5	13

Tabulation of the number of specific patterns associated with a given toxicity that: (1) have a confirmed match (see Section 2) with the metabolite profile of phenytoin treated animals at either the high or low dose, and (2) matched the metabolite profile of treated animals with a median correlation greater than 0.5 at either dose. Data taken from [25].

**Table 2:** Sum of toxicity patterns matching cyclosporin A-induced metabolite changes.

Toxicity	Cyclosporin A, low dose	Cyclosporin A, high dose
Blood, anemia		1
Bone, osteoblast inhibitor		2
GI tract, duodenum, iron deficiency	1	1
Immune system, immunosuppression	1	1
Kidney, diuretic effect		1
Kidney, interstitial nephritis		1
Kidney, glomerular tubular defect		1
Liver, paracetamol-like toxicity	2	2
Nervous system, serotonin receptor antagonist		1
Spleen, methemoglobinaemia		3

Grand total	4	14

See the legend for Table 1.

**Table 3:** Sum of toxicity patterns matching doxorubicin-induced metabolite changes.

Toxicity	Doxorubicin, low dose	Doxorubicin, high dose
Blood, anemia		2
Bone marrow, suppression	2	2
Hormones, antiandrogen		1
Systemic antiproliferative		1
Kidney, tubular toxicity	1	
Liver toxicity		1
Liver, enzyme induction		2
Thyroid, indirect effects		1

Grand total	3	10

See the legend for Table 1.

**Table 4:** Sum of toxicity patterns matching captopril-induced metabolite changes.

Toxicity	Captopril, low dose	Captopril, high dose
Adrenals, steroid biosynthesis inhibition		1
Bone marrow, suppression	2	
Kidney, glomerular tubular defect	1	3
Kidney, tubular toxicity	2	
Liver, oxidative stress	3	
Phthalate toxicity	1	

Grand total	9	4

See the legend for Table 1.

**Table 5:** Sum of toxicity patterns matching lisinopril-induced metabolite changes.

Toxicity	Lisinopril, low dose	Lisinopril, high dose
Blood, platelet aggregation inhibition		1
Kidney, ACE-inhibitor-like	1	1
Kidney, diuretic effect		1
Reduced food consumption	1	

Grand total	2	3

See the legend for Table 1.

**Table 6:** Summary of metabolite profiling and comparison with know toxicities.

Drug	Target	Rat study standard findings	Human clinical findings	MMtox prediction
Captopril	Kidney	Yes	Yes	Yes (∗)
Lisinopril	Kidney	Yes	Yes	Yes (∗)
Cyclosporin A	Kidney	Yes	Yes	Yes
Doxorubicin	Kidney	Yes	Yes	Yes
Phenytoin	Kidney	No	No	No
Captopril	Liver	No	Yes	Yes
Cyclosporin A	Liver	Yes	Yes	Yes
Doxorubicin	Liver	Yes	Yes	Yes
Lisinopril	Liver	No	No	No
Phenytoin	Liver	No	Yes	Yes
Cyclosporin A	Anemia	Yes	Yes	Yes
Doxorubicin	Anemia	Yes	Yes	Yes
Captopril	Bone marrow	Yes	Yes	Yes
Lisinopril	Platelet	Yes	Yes	Yes

*The treatments with these compounds were used to define some of the patterns for kidney toxicity.
